# Spatial and Temporal Characterization of Activity in Public Space, 2019–2020

**DOI:** 10.1038/s41597-022-01480-6

**Published:** 2022-07-05

**Authors:** Christa Brelsford, Jessica Moehl, Eric Weber, Kevin Sparks, Joseph V. Tuccillo, Amy Rose

**Affiliations:** Geospatial Science and Human Security Division, Oak Ridge National Laboratory, 1 Bethel Valley Road, Oak Ridge, TN 37830 USA

**Keywords:** Economics, Decision making

## Abstract

The data reported here characterize spatial and temporal variation in the ratio of short-to-long-duration visits in public places (i.e., points of interest) in the United States for each week between January 2019 and December 2020. The underlying data on anonymized and aggregated foot traffic to public places is curated by SafeGraph, a geospatial data provider. In this work, we report the estimated number and duration of “short” (i.e., <4 hours) and “long” (i.e., >4 hours) visits to public places at the US census block group level. Long visits are shown to be a good proxy for workers based on formal economic data. We propose that short visits are more likely to represent nonobligate activities: people visiting a public place for leisure, shopping, entertainment, or civic or cultural engagement. Our work constructs a ratio of short to long visits, which can be used to inform population estimates for nonworker use of public space. These data may be useful for understanding how people’s use of public space has changed during the COVID-19 pandemic and, more generally, for understanding activity patterns in public.

## Abstract (summary)

**Table:** 

Measurement(s)	Visits to a Public Venue
Technology Type(s)	mobile phone
Sample Characteristic - Organism	Homo
Sample Characteristic - Environment	public building
Sample Characteristic - Location	United States of America

## Background & Summary

The COVID-19 pandemic brought about profound change in the availability of mobile device–based mobility and behavioral information. Because these data can be used to estimate changes in social contact structure, people’s use of public space, or visitor population in a specific location, information derived from them can be put to lifesaving use in the context of emergency response, hazard mitigation, and public health. However, the extent to which these data represent real-world movement, behavior patterns, or other activities is still highly uncertain. In this paper, we create and present a dataset that builds a bridge between anonymized and aggregate mobile device visit data and formal economic and employment data. This allows us to estimate the real-world spatial distribution of nonworker visitors to public spaces, information that is not currently represented in any official statistics or census. These data may be useful for understanding how use of public space has changed during the COVID-19 pandemic and, more generally, for understanding activity patterns in public.

In this dataset, we use a Point of Interest (POI)–level dataset from SafeGraph https://www.safegraph.com, a geospatial data provider. SafeGraph’s weekly patterns data are curated from anonymized and aggregated device-level records of visits to public spaces and give an estimate of time spent in particular locations. SafeGraph then compiles the device level data into statistics on visits by POI^1^. We use these data to estimate the likely ratio of people engaged in nonobligate behavior in public spaces—shopping, leisure, entertainment and civic engagement—in comparison to workers there. We have produced the estimate reported here by summarizing the number and duration of “short” (i.e., <4 hours) and “long” (i.e., >4 hours) visits to POIs at the US census block group level. Our validation efforts show that long visits are a good proxy for the worker population in a given location. We propose that short visits are a good proxy for nonobligate activities performed by nonworker populations in a given location. We also describe the instantaneous ratio of short visits to long visits, which may be useful for estimating the nonworker use of public space. These data, reported at the US census block group level, by week, for 2019 and 2020, enable measurement of how spatial heterogeneity in the ratio of short to long visits in public spaces changed over 2019, a “baseline” year, and during 2020, a year profoundly influenced by the COVID-19 pandemic and numerous other natural disasters and extreme events.

## Methods

### SafeGraph Data: Collection and limitations

We use the Social Distancing Metrics previously published by SafeGraph during the COVID-19 pandemic to assess visits to census block groups. SafeGraph’s documentation on these data are available at https://docs.safegraph.com/docs/weekly-patterns. These data are currently available for academic, noncommercial use by filling out a data access request https://www.safegraph.com/academics. SafeGraph data are derived from a proprietary set of mobile device apps that record location information from the users on some schedule set by the apps themselves in a way that is consistent with the device technology, user settings, and the terms of use set by the device manufacturer and app developer. Location data are aggregated by SafeGraph to create an estimate of time spent in particular locations.

#### Device-level information

The SafeGraph data represent about 10% of the entire US population in any given week^1^. However, it is not known how a device ends up in the SafeGraph panel. There is no expectation that the panel represents the same individuals through time, or that the data panel captures all of a device’s locations. Devices drop in and out of the dataset depending on the user’s installation and use of the SafeGraph-partnering apps, as well as the app- and device-level settings on location services. Some devices may only appear in the panel when a particular app is actively being used, and some may appear at all times. As such, the raw data are simultaneously one of the most comprehensive sets of data that has ever been available on the location and behavior of individuals but also represent a deeply biased sample. Some aspects of this bias can be empirically addressed, and some cannot. It must also be noted that SafeGraph data represent the movement patterns of location-enabled devices, not individuals. Some people do not carry a smart device, some people carry multiple devices, some devices are shared across multiple individuals, and some people carry a smart device only some of the time. Challenges related to the data’s representativeness regarding movement and behavior patterns, generalization to the US population, and mechanisms by which mobile devices are either included or excluded must be addressed before the usefulness of SafeGraph data to determine the real-world use of public spaces can be fully evaluated.

One significant challenge that must be addressed in using SafeGraph data is the extent to which mobile device–level data should be inferred to represent comprehensive movement and behavior patterns for the individuals carrying the devices. SafeGraph does not release data that are resolvable to an individual device. We have no way of determining the share of public visits that are captured for an individual device. However, the data used in this work show a geographically diverse spike in visits that are recorded at UTC + 0. This 1-hour spike occurs across different time zones, and is at UTC + 0 in the United States regardless of whether the United States is on Daylight Savings time or Standard time. Depending on the geography, 50–75% more visits are recorded in this one hour than the hours on either side^2^. The visits recorded for this hour show the spatial distribution expected from visits recorded in the neighboring hours; they don’t appear to be randomly distributed or centered on different locations. We propose that this spike represents devices or apps that are set to “check in” only once per day, and the common default for this check-in is UTC + 0. The location information appears to be accurate, but the magnitude of the spike appears to show that we cannot consider the visit data to be comprehensive, even for devices in the panel.

The next major challenge involves determining the extent to which the individuals represented in the SafeGraph panel represent the US population as a whole, and the extent to which their behavior is representative of overall behavior. The mobile devices in SafeGraph’s panel are primarily smartphones. Smartphone device use is widespread in the United States: 80% of adults use a smartphone, but this ownership skews young, educated, wealthy, and urban^3^. Only 53% of US adults older than 65 used a smartphone in 2019, along with 66% of adults without a high school degree and 70% of people living in rural areas or with incomes less than $30,000 per year. Children are also far less likely to carry a smartphone than adults, and according to many interpretations of privacy law, should not be tracked at all. Because of the inherent exclusivity of smartphone use, the SafeGraph panel must be viewed as demographically biased.

Further, it is likely that the movement patterns of people who use devices that end up in the SafeGraph panel may be different from those of uncaptured devices. Devices in the SafeGraph panel may be more heavily used, have fewer privacy restrictions, spend more time in public, and exhibit other differences which cannot be measured from typical behavior patterns of the US adult population. We could use reweighting schemes to address underrepresented demographic categories, but these would not address the fundamental differences that determine an individual’s choice to carry a smart device or avoid its use as well as the individual’s choice of apps and privacy settings, which influence the probability of ending up in the SafeGraph panel.

These sampling inconsistencies limit the strength of inferences derived from these data, and any assessment should be made with caution. Nonetheless, the availability of SafeGraph data represents an increase of several orders of magnitude in the number of individuals whose behavior can be (imperfectly) observed. As such, they provide a valuable opportunity to observe changes in behavior and mobility patterns.

#### Point of interest (POI) data

The SafeGraph POI library represented nearly 5 million different POIs across the date range used in this study. POIs include commercial and industrial establishments, churches, schools, community centers, natural and civic landmarks, trailheads, beaches, and recreational venues. As such, the visits captured in these data potentially represent any activity in public spaces. SafeGraph’s advertisements claim they have “the most accurate POI and foot traffic data on the market.” While we do not seek to validate that claim, it suggests SafeGraph believes the strengths in their dataset lie in a relatively comprehensive set of POIs, and a data management system that captures visits to those POIs that are somewhat representative of real-world behavior patterns.

#### Visit data

The SafeGraph “weekly patterns data” are organized around weekly visits to one of the POIs in SafeGraph’s database. Each record in the weekly patterns data shows visits to one POI in one week and shows the total number of visits, median visit duration, and number of visits with durations of 0–4, 5–10, 11–20, 21–60, 61–120, 121–240, and > 240 minutes. Other information in the weekly patterns data which is not used for this paper includes mobility information such as an estimate of the home block group for visitors to the POI. A record is included in the database only if SafeGraph identifies four or more visits in that POI–week. An average of 260 million visits per week were recorded in the United States in 2019, approximately one for each US adult.

### Federally validated population, geographic, and employment data

#### US Census geographic data

SafeGraph assigns each of their POIs to a US census block group. US census geographies are freely available online at https://www.census.gov/geographies/mapping-files/time-series/geo/tiger-line-file.html. US census block groups change slightly each year to adjust for population growth and urbanization, and SafeGraph does not publish the vintage of their block groups. However, it is clear that the block groups SafeGraph uses come from 2019, before the major changes associated with the 2020 US census were available. There are 217,739 census block groups in the 2019 vintage of the US census for the 50 US states and Washington, DC.

#### Employment information

We validate aspects of the SafeGraph data–based short-to-long-visit ratio against the US Census Longitudinal Employer-Household Dynamics (LEHD) tables, particularly the LEHD Origin-Destination Employment Statistics (LODES) worker data^4^. For the work reported in this paper, we used the Workplace Area Characteristics LODES data table from 2018 at the block group level. These data represent the number of unique jobs in each block group for each of the North American Industry Classification System (NAICS) codes. We also used data from the US Bureau of Labor Statistics, provided by the Federal Reserve Bank of St. Louis (FRED)^5–13^, to measure average weekly hours of all employees by NAICS code. Combining LODES data with FRED data allowed us to estimate the average weekly hours per worker by block group, in addition to the LODES measure of total number of workers per block group.

#### Geographic validation data

For this work, we used a national building footprint dataset called USA Structures^14^ as an additional strategy for testing whether characteristics of the built environment such as the space devoted to office and retail are consistent with the visitation patterns implied by SafeGraph data. By intersecting the USA Structures data with parcel land use data^15,16^, we were able to evaluate the aggregate ground space dedicated to offices and retail uses at the census block group level. It should be noted that USA Structures data does not measure the height or number of stories per building and therefore is a less accurate measure in dense urban environments, where multistory buildings contribute to total building area that is quite different from a given building’s ground coverage.

### Analytical methods

To explore the extent to which SafeGraph data can be used to infer characteristics of real-world behavior patterns, we compared long-duration visits (i.e., >4 hours) to formal statistics on worker count, industry, and locations. If long-duration visits are a good proxy for worker population, we can use the ratio of short-to-long-duration visits to infer information about the location and population of nonobligate users of public space: people present in a specific public space for purposes of shopping, leisure, entertainment, or civic or cultural purposes. There are currently no reliable sources of information on the spatial allocation of this population segment.

We used SafeGraph data to estimate the short-to-long-visit ratio and average visit duration within US census block groups. The raw data can be aggregated to any higher-level geography and observed across different time scales. If paired with a spatially explicit measure of workers, the short-to-long-visit ratio may be useful for generating a more accurate and up-to-date measure of nonobligate users of public space than is currently available from any other source.

#### Measuring average visit duration for short and long visits

**Fig. 1:**
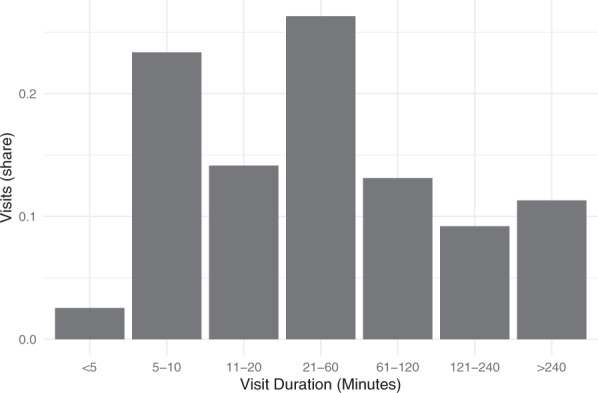
SafeGraph visits by visit duration, for a random sample of 500,000 POI-weeks from 2019. This shows the aggregate distribution of visit durations, according to the breakpoints available in the SafeGraph data.

SafeGraph reports visit duration information in two ways. They calculate median visit duration for all mobile device visits, and also classify each visit into one of seven bins of visit duration. These bins are 0–4, 5–10, 11–20, 21–60, 61–120, 121–240, and >240 minutes. Figure 1 shows the share of captured visits that falls into each of the visit duration bins. Data included are 500,000 randomly selected POI/week records from 2019. To estimate the average visit duration for short-duration visitors alone, we rely on the SafeGraph data reporting number of visits by binned visit duration. We assigned visit duration within each bin at the midpoint of that bin. The average visit duration for short visitors *ds*_*poi*_ is thus the average duration of the visits in bins less than 240 minutes.

There is no information in the SafeGraph data that can be used to inform visit duration for long visitors because we only know that the visit lasted longer than 4 hours. To assign a duration for long visitors, we relied on the hypothesis that long visitors are a good a proxy for workers. We assigned each long visitor a visit duration equivalent to their block group’s estimate of average worker shift length, as estimated from FRED and LODES data. We describe the duration of long visits (*dl*_*bg*_) at the census block group level rather than the POI level because this is the finest geographic scale for which data are available.

#### Measuring the instantaneous short-to-long-visit ratio

To enable population estimation, we estimated the instantaneous ratio of devices with long (>4 hours) and short (<4 hours) visit durations. We call this the short-to-long-visit ratio, *μ*_*slr*_. The instantaneous ratio provides an expectation of the proportion of people in a place by each visitor class at a particular moment. It is distinct from the total number of long or short visitors who may pass through over the course of a day. For example, if a fast food restaurant and a five-star restaurant have the same number of customers present at any moment over the course of the day, the fast food restaurant is expected to have served many more customers that day because people pass through it much more quickly. Visit duration is critical for making inferences about the population present in a specific location at any moment.

To estimate *μ*_*slr*_, we identified short visits, *sv*, and long visits, *lv*, from the number of visits of each class at the POI level. We then estimated the total short-visit minutes for each census block group: ${s}_{bg}=\sum \left(s{v}_{poi}\ast d{s}_{poi}\right)$. We estimated total long-visit minutes in an analogous way: ${l}_{bg}=\sum \left(l{v}_{poi}\ast d{l}_{bg}\right)$. In both cases, we summed over all POIs in the census block group to find total visitor minutes by short- or long-visit classification. Finally, the instantaneous ratio of short to long visitors is ${\mu }_{slr}=\frac{{s}_{bg}}{{l}_{bg}}$. The ratio is dependent on the assumed duration of long visits and thus relies on our hypothesis that long visitors are representative of workers.

**Fig. 2:**
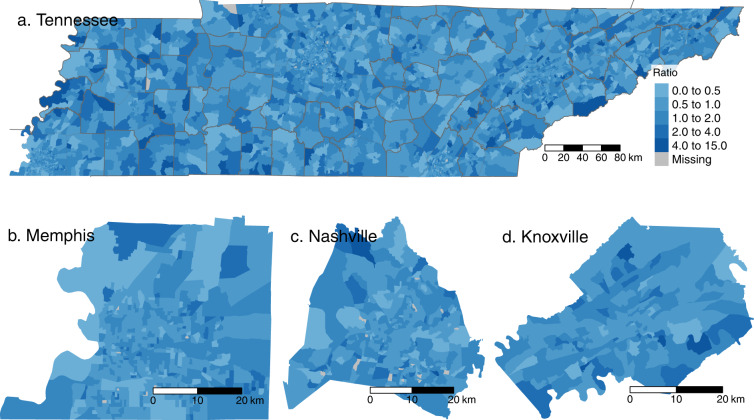
Census block group level short-to-long-visit ratio for Tennessee in 2019. Nationwide data is available in the full data files. There is substantial spatial heterogeneity within cities and across the state. This suggests spatial variation in the balance between workers and visitors across different places.

**Fig. 3:**
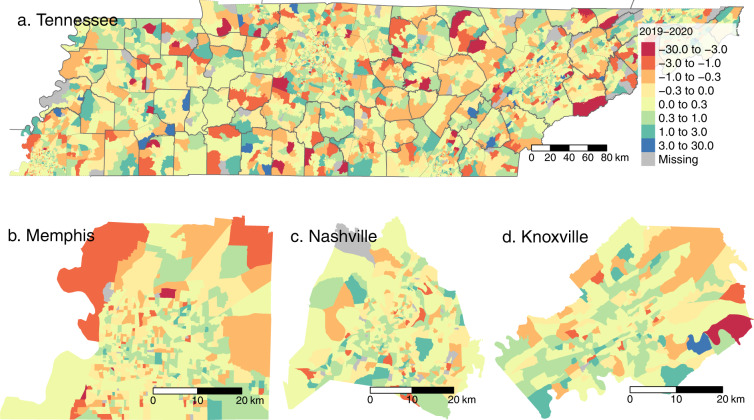
Difference between census block group level short-to-long-visit ratio from the US period of lowest mobility during the COVID-19 pandemic (March 15, 2020–April 8, 2020) and the 2019 average short-to-long-visit ratio. It appears that the short-to-long-visit ratio increased in urban centers, particularly in retail-dominated locations, while it decreased in suburbs and exurbs. This would imply decreases in short visits in urban centers, and either increases in visits or decreases in workers in more outlying areas.

The short-to-long-visit ratio is estimated for every census block group in each of the 50 states and the District of Columbia by week. There are gaps in the data where SafeGraph did not record both short and long visits in a particular block group–week. To demonstrate some of the spatial variability in these data, Fig. 2 shows the short-to-long-visit ratio by census block group for Tennessee and for Tennessee’s three most populous counties, based on 2019 visit data. Figure 3 shows the difference in short-to-long-visit ratios by census block group between the height of COVID-associated mobility restrictions (March 15 to April 8, 2020) and the 2019 average. The maps show that some of the largest reductions in the short-to-long-visit ratio in the early COVID shelter-at-home period occurred in places with unusually high short-to-long-visit ratio in 2019, and in places dominated by shopping facilities.

#### Aggregation to higher-level geographies and coarser temporal resolution

To aggregate from the weekly census block group level short-to-long-visit ratio produced for this data descriptor to broader geographies or longer time periods, we calculated the total short- and long-visit times across all block groups within the focal geography and all weeks within the focal time period.

1 $${\mu }_{slr}=\frac{\sum {s}_{bg}}{\sum {l}_{bg}}$$

**Fig. 4:**
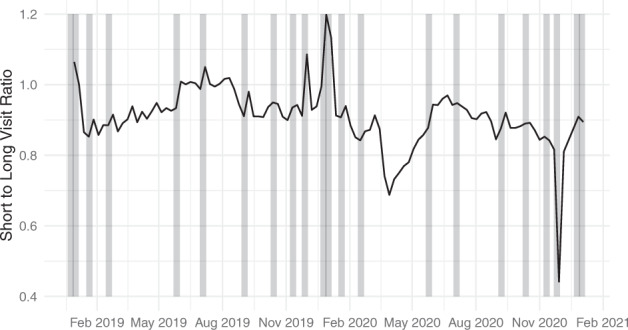
Time series of the national average short-to-long-visit ratio by week for the full study period. Background grey bars show the ten United States Federal Holidays. We see a dramatic drop in the short-to-long-visit ratio from mid-March 2020 to July 4th 2020. It’s also clear that the behavior of the short-to-long-visit ratio during the 2020 winter holiday season differs markedly from the 2019 holiday season. While 2019 saw dramatic increases in short-to-long-visit ratio for Thanksgiving, Christmas, and New Years, 2020 saw the opposite. This coincided with the United States’ first truly national scale COVID-19 wave.

Table 1 shows the short-to-long-visit ratio, by percentile, for a range of different levels of geographic aggregation from the 2019 table data. At the census block group level, overall short-to-long-visit ratio ranges from 0 to 41 shoppers per worker, with the 10th and 90th percentile being 0.49 and 1.71, respectively. At higher levels of geographic aggregation, the extreme values are absorbed into the broader average. When the 2019 short-to-long-visit ratio values are aggregated to counties, the 10th and 90th percentile short-to-long-visit ratio values are 0.68 and 1.08, respectively, while the extreme values are 0.33 and 1.95. For the purpose of exploring temporal patterns in short-to-long-visit ratio during the 2020 COVID-19 waves, we calculate the national average short-to-long-visit ratio by week for our full time series, presented in Fig. 4. We see a significant decline in short-to-long-visit ratio during the maximal period of COVID-19 behavioral change, rebounding to near-previous levels by July 4th 2020. This implies that there disproportionately fewer short visits than long visits the period of greatest COVID-19 associated movement reduction; consistent with a pattern of dramatically reduced shopping, leisure and entertainment activities, while workplace activities were less curtailed.

**Table 1:** Actual short-to-long-visit ratio percentiles (not-weighted by population) across the geographic scales of analysis.

Percentile:	Min	1%	5%	10%	50%	90%	95%	99%	Max
Block Group	0	0.28	0.41	0.49	0.88	1.71	2.12	3.32	40.90
Tract	0	0.36	0.48	0.55	0.88	1.49	1.76	2.42	31.81
County	0.33	0.55	0.63	0.68	0.87	1.08	1.14	1.29	1.95
Metro. Stat. Area	0.63	0.71	0.76	0.80	0.94	1.10	1.14	1.28	1.45

## Data Records

The data records produced for this work are available at 10.6084/m9.figshare.c.5656993.v1^17^. The data records in this table are stored in two csv files, one for 2019 and one for 2020, and a supplementary SQL script which demonstrates how the files were produced from raw SafeGraph data. The structure for both files is shown in Table 2. The files include weekly records by census block group for weeks ending January 1, 2019, to December 31, 2020. Each row uniquely identifies one census block group–week combination.

**Table 2:** Sample of the data described in this data descriptor paper.

geoid	week_ending_date	n_visits	n_poi	short_visits	short_minutes	long_visits	long_minutes	slr	mean_short_dwell	mean_long_dwell
010010201001	2019-12-29	419	8	380	17467.5	39	15593.13	1.12	45.97	399.82
010010201002	2019-12-29	136	4	109	9582.5	27	11189.70	0.86	87.91	414.43
010010202001	2019-12-29	116	6	99	4607.5	17	7235.05	0.64	46.54	425.59
010010202002	2019-12-29	2494	47	2376	120260	118	46687.16	2.58	50.61	395.65
010030101001	2019-12-29	13	2	13	730	0	0.00		56.15	
010030101002	2019-12-29	567	17	560	13117.5	7	3065.07	4.28	23.42	437.87
010030101003	2019-12-29	355	9	350	10765	5	2091.28	5.15	30.76	418.26

Note: For presentation here, data are rounded to two decimal places. Data are represented with full precision in the full dataset.

In 2019, SafeGraph reported visits to POIs in 213,386 of the 217,789 census block groups. Our data are not in balanced panels: rows are excluded when SafeGraph did not report any data for that block group–week combination. The data are ordered first by the 12-digit US census geographic identifier code used to for census block groups and then by date. There are 21,863,582 rows in the two year dataset.

Table 2 shows the structure of the included dataset. The columns are defined as follows:

- “geoid” shows the 12-digit US census block group geographic identifier (*i*).
- “week_end_date” shows the end date for the included data (*t*). Data are reported weekly.
- “n_visits” shows the total number of unique visits recorded in *it*.
- “n_poi” shows the number of POI weeks with visits in the data in *it*.
- “short_visits” shows the number of visits with a duration of less than 4 hours.
- “short_minutes” shows the aggregate duration of visits lasting less than 4 hours.
- “long_visits” shows the number of visits with a duration of more than 4 hours.
- “long_minutes” shows the aggregate duration of visits lasting more than 4 hours.
- “slr” shows the mean ratio of short-to-long-duration visits for all recorded visits in *it*. This is the short-to-long-visit ratio.
- “mean_short_visit” shows the estimated average visit duration for short visits to all POIs in *it*.
- “mean_long_visit” shows the estimated average visit duration for long visits to all POIs in *i*. Note that this variable is not time dependent.

The short-to-long-visit ratio does not have a finite value for place–date combinations when zero long visits are recorded. In this case, we put NULL values in the short-to-long-visit ratio column, which are represented as blanks in the CSV. For this reason, the most accurate strategy for spatial or temporal aggregation is to sum the total minutes for short and long visits over the aggregation scope, and then divide short minutes into long minutes.

## Technical Validation

These data are impacted by several key sources of errors, uncertainty, and bias. We know that devices captured in the SafeGraph panel are not representative of all public activity. It is very likely that inclusion in the SafeGraph dataset is based on nonrandom behavioral characteristics. There is demographic heterogeneity in device use, which may lead to differential capturing of long vs. short visitors, which would bias the accuracy of *μ*_*slr*_. The most problematic bias for our work is spatial inconsistency in how short- and long-duration visits are captured.

Validation of these data is challenging because there are no formal, national-scale sources of information on movement and behavior patterns for people who are engaged in nonobligate activities. This section outlines several strategies for assessing the internal and external validity of the components of the short-to-long-visit ratio. First, we compare the number of long-duration visits to census-derived employment statistics. Second, we explore differences in correlations between components of the SafeGraph visit data, employment statistics, and land use data. Third, we compare the SafeGraph estimates of visitors and workers and their ratios to real-world data gleaned from tax filings and organizational self-reports for zoos across the country. Most zoos, as nonprofit organizations, are required to report their employee numbers in annual tax documents. They also typically report annual visitor data in the same documents. This gives us a direct (although small-scale) comparison point for the SafeGraph visit data and an alternative estimate of *μ*_*slr*_.

While these validation strategies are imperfect, we propose that they present a picture that consistently shows significant noise in the SafeGraph data and enough signal in the data to confirm they can be used to infer characteristics of real-world behavior.

### Comparing safegraph long-duration visits to lodes workers

**Fig. 5:**
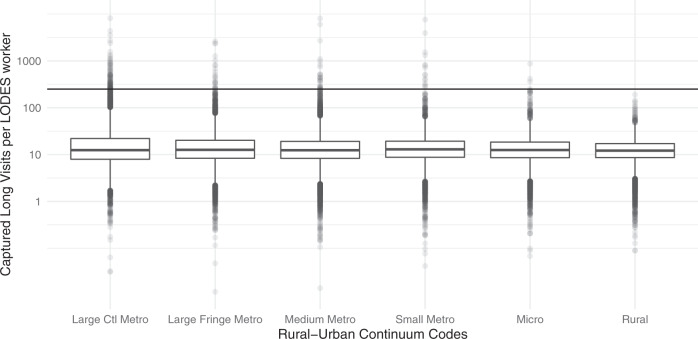
Number of captured long visits in 2019 per LODES worker, aggregated to census tracts, and split by urban classification^18^. The bold horizontal line at 250 shows the estimated annual workplace visits for a full time worker. We see there is no significant difference in the long visits to worker relationship based on urban classification.

To test whether long-duration visits are a good proxy for workers, we compared SafeGraph-captured long visits to the number of jobs recorded in the LODES dataset. We found, on average, 10.57 long visits are captured for each job reported in the LODES dataset. For comparison, if all workers went to work 5 days per week, 50 weeks per year, there would be 250 workplace visits for each worker. If long visits are a good proxy for workers, this suggests the SafeGraph panel captures about 4 percent of worker visits. Figure 5 shows that there is no detectable geographic bias in the relationship between long visits and workers from the perspective of urbanization levels. This figure segregates census block groups into the six urban–rural classes defined by Ingram and Franko^18^ and identifies the number of captured long visits for each tract worker by urban class. The median number of visits per worker varies from 12.2 (rural areas) to 12.9 (small metro areas) for each of the six urbanization classes. The average number of visits per worker by urban class ranges from 14.2 (rural) to 22.5 (large central metro). Neither the mean nor the median shows statistically significant differences across classes.

We next compared the number of long visits to LODES workers within each census block group in a continuous way. We used a log-log regression to explore percent change in the relationship between long visits and LODES workers.

We observed heteroskedasticity in the noise for the long-visit data, pointing to diminished accuracy in both long-visit counts and worker counts in areas with small worker totals. This problem results in part from anonymization of public-use Longitudinal Employer-Household Dynamics (LEHD) releases from which LODES files are derived. To balance statistical accuracy with nondisclosure of employee–employer relationships, LEHD releases are noise infused such that files with lower structural complexity resemble the unprotected (i.e., restricted-use) files less so than ones with higher complexity^19–22^. Additionally, worker counts are suppressed for employers below a threshold number of employees^21^. The SafeGraph data also are likely to have diminished accuracy in block groups with few visits. SafeGraph excludes POIs from the weekly patterns data when there are fewer than four visits to that POI. Census block groups that contain few POIs are therefore more likely to be excluded from the dataset and are more likely to have inaccurate visit estimates.

Given these issues, we chose to examine the relationship between SafeGraph and LODES using total least squares regression instead of ordinary least squares. Total least squares finds the best fit line to minimize the total Euclidean distance from the best fit line to all points rather than the y-direction distance. This creates a symmetrical treatment of the x and y variables and reduces the effect of noise in both variables. This regression was estimated on data aggregated to the census tract scale and included all known visits in 2019. The total count of 2019 visits in a census tract ranges from 4 to 33.1 million. To address the wide range in visits by census tract, each observation was weighted by the total number of visits of all durations captured by SafeGraph for that tract in 2019. Numerically, to set the intercept to zero for a total least squares calculation, we demeaned both the long visit and worker data. Setting *w* as the worker count and *lv* as the number of long visits, and using $\bar{w}$ and $\bar{l}v$ to denote mean workers and long visits, we estimated:

$$log\left(\frac{lv}{\bar{l}v}\right)=\beta log\left(\frac{w}{\bar{w}}\right).$$

**Fig. 6:**
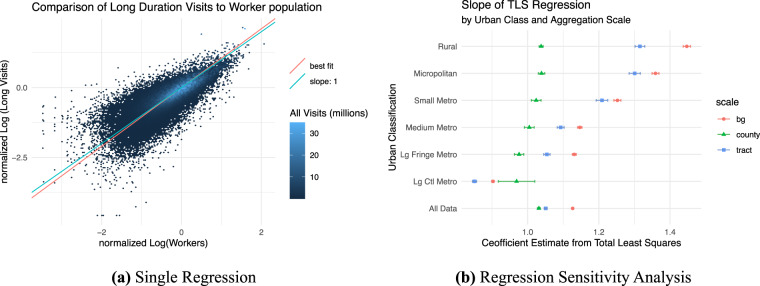
In (**a**) we compare the normalized log of LODES measured workers to the normalized log of SafeGraph estimated long visits. These data include all visits in 2019, and are aggregated to the census tract. For the purpose of visualization, a hex plot is used to show the data, with the sum of both long and short 2019 visits used as the metric for color intensity. The pink line shows the estimated best fit, while teal shows a slope of 1 for comparison. In (**b**), we perform a regression sensitivity analysis which shows the slope of the best fit line for several different regression strategies: aggregating the data to the block group, tract, and county spatial scale, and segmented regressions within each of the six urban classes, in addition to a regression including all data. In all cases, the data includes 2019 visits only.

Figure 6(a) shows the visit data, the estimated best fit line, and a line with slope 1 for reference. The data are presented as a hex plot, where each area is colored by the total number of visits to all census block groups with that combination of measured workers and visits. We found that $\widehat{\beta }$ = 1.05, with a standard error of 0.002. If both data sources were unbiased and long visits are a good proxy for workers, we would expect that *β* = 1. This result implies that census block groups with a 1% greater worker population can be expected to have a 1.05% greater SafeGraph estimate of long-duration visits, suggesting that SafeGraph captures a larger share of worker visits in places with more workers. This may be explained by the fact that more densely used spaces typically have more robust communication infrastructure and so may better capture worker visits. As a robustness check on these regressions, we performed the same analysis on several subsets of the data and across different geographic aggregation scales. We aggregated POI visit information to the census block group, tract, and county levels. We also separately tested the *lv*-*w* relationship within each of the six urban classes, in addition to a comprehensive dataset. Figure 6(b) shows the regression coefficient *β* and the 95% confidence interval for each regression. These results show that SafeGraph may undercount the number of worker visits in locations with few workers overall and does so with greater bias at small geographic scales and in less-urban environments. However, we believe these results show that long visits can be used as a reasonable proxy for worker population, as long as the data are not split too finely.

### Correlation of components

Next, we explored the differences in correlation among combinations of components of the SafeGraph data and the available large-scale data for validation (i.e., employment statistics and land use characteristics). The components we explored are total, short, and long visits in the SafeGraph data, LODES measured worker population, and retail and office space from the USA Structures data. We expected workers to be more closely correlated with long visits than with short visits. We expected short visits to be more closely correlated with retail space than office space. We expected both long visits and workers to be more correlated with office space than retail space.

**Fig. 7:**
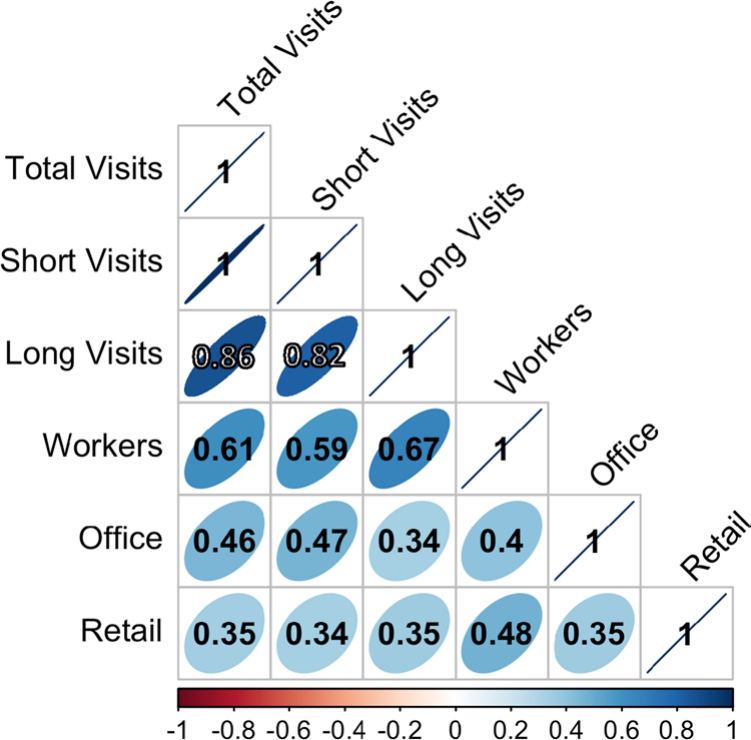
Correlation matrix for tract-level data for 2019 visits. We see that the correlations across SafeGraph data (total, short, and long visits) are the strongest overall correlations. The strongest correlation between any SafeGraph based metric and the economic data is between workers and long visits, which suggests that long visitors are a reasonable proxy for workers.

Figure 7 shows a summary correlation plot for all combinations of these data components. SafeGraph data include all visits in 2019. The tightest observable correlations are within the SafeGraph data: long, short, and total visits. Because of this tight correlation across the SafeGraph data components, the capacity for differential comparisons with our validation data sources is weak. Nonetheless, there is a stronger correlation between LODES workers and long visits than between workers and short visits, as expected.

The correlations on office space and retail space are weaker than for the other data components and, in general, are not consistent with our expectations. Office space is more closely correlated with short visits than long visits, while retail space is very slightly more closely correlated with long visits than short visits. Notably, workers are also more closely correlated with retail space than office space. This suggests that measures of ground area devoted to a particular purpose may not be a useful measure of the population there, when observed at a national scale.

The unexpected correlations between visitor count and measures of office or retail space could partially result from complex or nonlinear relationships between urban density, building size and height, and visit patterns. USA Structures data do not include building height, it only describes land surface area dedicated to office or retail activities. Office spaces are more likely to be in multistory buildings than retail spaces, and the space available per office worker may also be more dependent on urban density than the same calculations in a retail context. People may be more likely to visit office or retail spaces in dense urban environments, where the space available is more constrained.

### Component comparison: POIs

We also attempted to manually validate SafeGraph data for several POIs for which we can find real-world estimates of workers and visitors. Nonprofit (i.e., 501(c)3) organizations report their number of employees on Form 990, a publicly reported tax document they are required to file. Many institutions with a public education or public activity mission also voluntarily report their annual number of visitors. Zoos are one such POI category that report both worker and visitor numbers, and they have a place-based mission: engagements with the facility are likely to be physically located at the facility (e.g., elephants don’t go to visit schools). Other educational spaces such as nonprofit museums typically combine reporting of visitors to the museum and participants of their outreach activities, so it is more difficult to estimate the actual number of visitors to the physical location. Table 3 shows the available information on 10 single-location zoos from across the United States. Worker numbers were taken from the 2019 fiscal year based on the zoo’s Form 990, which was accessed from an online data repository for nonprofit information (https://causeiq.com). Visitor counts are identified from documentation on Form 990 or by manually searching the zoo’s website and publicity materials if visitor counts are not included in Form 990. To infer a Form 990–based short-to-long-visit ratio, we assumed visitors averaged a 2-hour visit duration and employees averaged 0.65 of an FTE (1,347 hours) over the course of 2019. This worker duration is equivalent to 5.2 hour shifts on a 5-day per week schedule, the average work hours reported in the FRED data for employees in Leisure and Hospitality, the appropriate classification for zoos^12^. Table 3 also presents near-equivalent SafeGraph measures.

**Table 3:** Facility-level validation comparing publicly reported information from Form 990 to SafeGraph reported data for representative US zoos.

Facility	990:Visitors	990:Employees	990:SWR	SG:Shopper Hours	SG:Worker Hours	SG:SWR
San Diego Zoo	5,500,000	4,032	2.0	476,848	179,465	2.7
Houston Zoo	2,550,453	675	5.6	7,441	300	24.8
Denver Zoo	1,900,000	606	4.6	9,465	1,450	6.5
Phoenix Zoo	1,435,070	749	2.8	4,713	2,345	2.0
Grassmere Nashville Zoo	1,266,764	371	5.1	78,094	53,685	1.5
Philadelphia Zoo	1,200,000	638	2.8	52,103	26,885	1.9
San Francisco Zoo	1,000,000	258	5.7	37,110	16,580	2.2
Zoo Atlanta	990,000	454	3.2	4,712	4,600	1.0
Tulsa Zoo	600,000	254	3.5	6,496	1,370	4.7
Zoo Knoxville	453,924	350	1.9	11,097	4,510	2.5
mean	1,689,621	839	3.0	68,808	29,119	2.7

Note: 990 data is from the 2019 fiscal year, with visitor data from the year nearest to 2019 with reported information. SafeGraph data covers the 2019 calendar year. In all cases, data reflect the pre–COVID-19 period.

The visit-weighted average short-to-long-visit ratio for all zoos is similar as shown in the Form 990 and SafeGraph data. For reference, the overall average for zoo short-to-long-visit ratio s is in the 95th percentile for census block group level POI short-to-long-visit ratio s. This seems consistent: zoos are generally a high-throughput activity, with little direct interaction between visitors and staff. The correlation between the Form 990 and SafeGraph data for employees is 0.86 and for visitors is 0.79, and for the short-to-long-visit ratio it is 0.23. The high correlation values for workers and visitors are in part driven by the very large differences in the sizes of zoos; the correlation across short-to-long-visit ratio s is much lower because the range is smaller. Note that the Houston Zoo has both anomalously few SafeGraph recorded visitors, and an extremely high short-to-long-visit ratio.

These validation exercises suggest that while the SafeGraph data are an imperfect measure of real activity in public spaces, they appear to be generally representative of real-world processes.

## Usage Notes

Data is available at 10.6084/m9.figshare.c.5656993.v1 This repository also includes an SQL file demonstrating how these tables are generated from the raw SafeGraph data, after it has been read into a database for processing.

## Data Availability

The code used to generate the main data table is available at 10.6084/m9.figshare.c.5656993.v1. The SQL script included with the data DOI demonstrates the steps that we took to produce this table from SafeGraph’s raw data after it has been read into a database for processing.
